# Benefits of enhanced recovery after surgery in robotic nephrectomy

**DOI:** 10.1007/s00345-025-06131-0

**Published:** 2025-12-23

**Authors:** William Pierre Schrock, Jason M. Farrow, Kevin M. Backfish-White, Amanda Marinho Lima, Sydney Elizabeth Strup, Jiangqiong Li, Chandru Sundaram, Amy L. McCutchan

**Affiliations:** 1 Department of Urology, Indiana University School of Medicine, Indianapolis, IN USA; 2 Department of Anesthesiology, Indiana University School of Medicine, Indianapolis, IN USA; 3 Department of Biostatistics, Indiana University School of Medicine, Indianapolis, IN USA

**Keywords:** Enhanced recovery, Robotic surgery, Nephrectomy

## Abstract

**Purpose:**

Enhanced recovery after surgery (ERAS) is an evidence-based perioperative care approach aimed at attenuating surgical stress response and facilitating patient recovery. This study compared postoperative outcomes between an ERAS perioperative model to a traditional, unstandardized perioperative care practice in patients undergoing robotic nephrectomy.

**Materials and methods:**

A total of 206 patients who underwent robotic renal surgery were stratified into traditional and ERAS cohorts. In total, 111 patients received the ERAS pathway, and 95 received traditional care. Data was collected through a retrospective review of electronic medical records. The primary outcome was length of hospital stay (LOS). The secondary outcomes included patient recovery milestones, pain scores, opioid use, patient complications, 30-day readmission rates, incidence of surgical site infection, and total hospital costs.

**Results:**

Implementation of the ERAS pathway was associated with shorter hospital stay (median LOS 2.2 days vs. 2.3 days *p* = 0.011), lower post-operative pain scores and lower total opioid requirements at all analyzed time points (0–1, 1–24, and 24–48 h). No statistically significant differences were observed in adverse events, rates of ileus, time to first flatus, surgical site infection, or oral intake. Hospital costs were similar between groups. 30-day readmission was higher in the traditional care cohort (9% vs. 2% *p* = 0.035).

**Conclusions:**

ERAS was associated with reduced length of hospital stay, improved pain scores, reduced opioid use, and lower incidence of hospital readmission in patients undergoing robotic nephrectomy.

## Introduction

Hospital discharge after surgery can prove to be troublesome due to factors such as the need for parenteral analgesia, gut dysfunction, and lack of mobility. Enhanced recovery after surgery (ERAS) is a perioperative care approach that standardizes and integrates the best evidence-based perioperative practices to attenuate surgical stress response and facilitate patient recovery [1]. Pathways are based on a multidisciplinary, patient-centered approach and typically include pre-, intra-, and postoperative elements, such as patient counseling, exercise, immune-nutrition, carbohydrate loading, and multimodal pain management strategies with early ambulation and feeding. ERAS pathways have been developed in many surgical subspecialties, and have been shown to reduce patient morbidity, hospital costs, and length of hospital stay across several patient populations.

Within the practice of urology, a growing body of literature suggests ERAS pathways are of benefit to patients undergoing radical cystectomy in reducing the length of hospital stay (LOS), major complications rates, time to ambulation, and return of bowel function [2]. Additionally, ERAS appears to confer benefits in patients undergoing minimally invasive urologic surgeries such as robotic/laparoscopic prostatectomy, adrenalectomy, and cystectomy resulting in similar reductions in LOS, complication rates, and total costs [3–5]. In patients undergoing minimally invasive living donor nephrectomy, ERAS pathways have been shown to decrease LOS as well as postoperative opioid requirements without impacting readmission rates or complications [6, 29].

Despite its implementation in various minimally invasive urologic surgeries, little is known about the impact of ERAS in patients undergoing robotic nephrectomy for malignant and nonmalignant renal pathology. Currently, most renal surgeries are performed robotically, and historical data show lower perioperative complication rates and shorter hospital stays than traditional open surgery ([7]– [8]). However, patients undergoing minimally invasive procedures continue to experience barriers to discharge including incisional pain and abdominal discomfort or distention, which may be ameliorated by implementation of ERAS protocols. The primary objective of this study was to evaluate the impact of a comprehensive, standardized ERAS pathway in patients undergoing robotic nephrectomy compared to that in patients receiving traditional non-standardized care.

## Materials and methods

### Study design

There was no funding for this project, and the authors did not have affiliations in any organization or entity with any financial interest in the subject matter or materials discussed in this manuscript. ALM, CPS contributed to the design and implementation of the research. WPS, JMF, KMB, AML, SES, and EJ contributed to the analysis of the results and to the writing of the manuscript. SES is the corresponding author. ALM conceived the original and supervised the project.

After approval by the Indiana University Medical Center Institutional Review Board (IRB No.10768), perioperative data were collected for patients who underwent robotic partial, simple, or radical nephrectomies for the years preceding ERAS implementation (December 2016 to December 2017, traditional care) and for the years following ERAS implementation (June 2018 to November 2020, ERAS pathway). Human Ethics and Consent to Participate declarations: not applicable. A 6-month wash-in period was included between the pre-ERAS and post-ERAS cohorts to account for training and familiarity with the ERAS pathway. The primary outcome was the hospital LOS. The secondary outcomes included postoperative pain scores and opioid use, time to oral liquid and solid food intake, time to flatus, ileus, nausea, 30-day readmission rate, surgical site infection rate, patient complications, and total hospital costs.

Prior to the introduction of the ERAS pathway at our institution, there was no standardization in the perioperative care of patients undergoing robotic nephrectomy, and patients were cared for according to physician preference. The nephrectomy ERAS pathway was modified from our institution’s colorectal surgery and cystectomy ERAS pathways.

### ERAS pathway

The ERAS pathway used in this study is described below *(*Table 1*)* and now represents a standard of care for all patients undergoing nephrectomy at the Indiana University Medical Center (IUMC). Patient care was coordinated by a multidisciplinary team of nurses, anesthesiologists, and surgeons until discharge.

**Table 1:** ERAS pathway for robotic nephrectomy at Indiana university

*Preoperative Management*
Preadmission counseling and medical optimization
ERAS pathway education
Physical exercise and smoking cessation encouragement
Immunonutrition with Impact Advanced Recovery
Hibiclens wash & mupirocin 1 day before surgery and morning of surgery
Carbohydrate drink loading with Gatorade G series
Oral analgesics administration with acetaminophen (1000 mg), gabapentin (600 mg, 300 mg for patient’s > 70)
*Intraoperative Management*
Pre-incisional quadratus lumborum block or 4 quadrant transversus abdominis plane block or post-incisional surgical site local infiltration with 266 mg of liposomal bupivacaine (Exparel^®^).
Opioid sparing technique with subanesthetic ketamine dosing.
Targeted goal-directed fluid management
All patients received at least two first-line antiemetics. Patients with >2 risk factors for PONV received additional antiemetics
Lung-protective mechanical ventilation
Removal of nasal or oral gastric tube before extubation
*Postoperative Management*
Oral liquids on POD0, regular diet on POD1
IV fluids discontinued once oral intake achieved
Incentive spirometry encouragement
Day of surgery ambulation
Scheduled adjunctive analgesics (PO gabapentin 300 mg at night, PO acetaminophen 1000 mg every 8 h)
Oxycodone or tramadol PO as needed
Rotational multimodal antiemetics as needed
DVT prophylaxis for simple, radical nephrectomies, none for partial nephrectomies
Bladder catheter removed POD1

### Data collection and statistical methods

Data was collected through a review of the electronic medical record system (Cerner Millennium). Specifically those patients who underwent robotic partial, simple, or radical nephrectomy at the IUMC. Propensity score matching was used to minimize baseline differences and reduce potential confounding. The propensity score was estimated using a logistic regression model, with the status of traditional care or ERAS pathway as the outcome and a set of demographics (i.e., age, sex, BMI, smoking status, diabetic status) and surgical characteristics (i.e., ASA status, surgery duration, robotic nephrectomy type) as covariates. Propensity score matching yielded a total of 95 matched pairs (Tables 2 and 3). After matching, statistical comparison of postoperative outcome variables were conducted within matched dataset: numerical variables were analyzed using pairwise Wilcoxon signed-rank test, while categorical variables were evaluated using McNemar’s test.

**Table 2:** Demographics and surgical characteristics before propensity matching

Variable	ERAS (*n* = 111)	Traditional (*n* = 95)	MSD (before match)
Age, mean (sd)	62.2 (13.1)	60.7 (13.1)	−0.111
Age70: yes, n (%)	31 (30%)	22 (20%)	−0.113
Sex: Female, n (%)	47 (40%)	42 (40%)	0.038
Sex: Male, n (%)	64 (60%)	53 (60%)	−0.038
BMI, mean (sd)	29.3 (5)	29.8 (5.9)	0.086
ASA: 1, n (%)	1 (0%)	1 (0%)	0.015
ASA: 2, n (%)	21 (20%)	28 (30%)	0.232
ASA: 3, n (%)	86 (80%)	62 (70%)	−0.257
ASA: 4, n (%)	3 (0%)	4 (0%)	0.075
Surgery_Duration_min, mean (sd)	187.7 (48)	191.4 (65.3)	0.056
Robotic_nephrectomy_type: partial, n (%)	69 (60%)	67 (70%)	0.183
Robotic_nephrectomy_type: radical, n (%)	36 (30%)	25 (30%)	−0.139
Robotic_nephrectomy_type: simple, n (%)	6 (10%)	3 (0%)	−0.129
Smoker: Yes, n (%)	47 (40%)	27 (30%)	−0.309
Diabetic: Yes, n (%)	21 (20%)	23 (20%)	0.124

**Table 3:** Demographics and surgical characteristics after propensity matching

Variable	ERAS (*n* = 95)	Traditional (*n* = 95)	MSD (after match)
Age, mean (sd)	62.2 (13.4)	60.7 (13.1)	−0.112
Age70: yes, n (%)	26 (30%)	22 (20%)	−0.1
Sex: Female, n (%)	42 (40%)	42 (40%)	0
Sex: Male, n (%)	53 (60%)	53 (60%)	0
BMI, mean (sd)	29.6 (4.9)	29.8 (5.9)	0.046
ASA: 1, n (%)	1 (0%)	1 (0%)	0
ASA: 2, n (%)	20 (20%)	28 (30%)	0.185
ASA: 3, n (%)	71 (70%)	62 (70%)	−0.199
ASA: 4, n (%)	3 (0%)	4 (0%)	0.052
Surgery_Duration_min, mean (sd)	190.9 (47.8)	191.4 (65.3)	0.007
Robotic_nephrectomy_type: partial, n (%)	63 (70%)	67 (70%)	0.092
Robotic_nephrectomy_type: radical, n (%)	29 (30%)	25 (30%)	−0.096
Robotic_nephrectomy_type: simple, n (%)	3 (0%)	3 (0%)	0
Smoker: Yes, n (%)	35 (40%)	27 (30%)	−0.187
Diabetic: Yes, n (%)	20 (20%)	23 (20%)	0.074

For postoperative variables, pain scores were assessed using a 0–10 visual analog scale (VAS) as per nursing routine. The highest pain reported each day was recorded from the day of surgery until discharge or until the 48-hour mark postoperatively, whichever occurred first. Postoperative IV and oral opioid administration was standardized using oral morphine equivalents (OME). Postoperative opioid administration was recorded and summed at 1, 24, and 48 h after arrival in the postoperative anesthesia care unit and tested for statistical significance. The patient’s total opioid requirements for 48 h postoperatively were also analyzed. Readmissions were defined as any admission to the IUMC or associated hospitals for any reason within 30 days after discharge. Any surgical site infection was documented for up to 30 days after discharge. Postoperative complications included patients who experienced pneumonia, respiratory failure, stroke, cardiac arrhythmia, heart failure, myocardial infarction, deep vein thrombosis, pulmonary embolus, urinary tract infection, acute kidney injury, dialysis, sepsis, and delirium. The total patient hospital cost data were collected and obtained from the IUMC Revenue Department.

Matched data returned 190 patients for consideration (95 pairs of two pathway types). For continuous outcome measures, some paired observations were removed due to missing data. All outcomes were analyzed using 95 pairs of data except for time to 1 st oral liquid intake (89 pairs), time to 1 st oral solids (45 pairs), time to flatus (52 pairs) and total hospital costs (90 pairs). For pain score measures, all measures were analyzed using 95 paris of data, except the highest pain score during stay (94 pairs) and the highest pain score during 24 to 48 h (89 pairs). For OME measures, all measures were analyzed using 95 pairs of data except the OME during 24 to 48 h (94 pairs).

## Results

After propensity score matching, for the primary outcome, i.e., hospital length of stay (LOS, days), paired Wilcoxon signed rank test showed a p value = 0.011 indicating a statistically significant difference in hospital length of stay.

No statistically significant difference was detected between the cohorts in terms of time to oral solid intake, oral liquid intake, time to first flatus, rates of postoperative ileus, nausea and vomiting, or other postoperative complications. Both cohorts had a similar incidence of surgical site infection within 30 days of discharge. Total hospital costs were similar between groups. Patients undergoing ERAS protocol had a lower rate of 30-day readmission (2% vs. 9% *p* = 0.035) (Table 4).

**Table 4:** Postoperative patient Outcomes

Outcome		ERAS Pathway	Traditional care	*P*-value	Median difference (ERAS – Traditional) or Odds ratio	95% CI
Hospital Length of Stay (LOS, days)		2.2 (1.7–2.4)	2.3 (2.1–3.2)	**0.011**	−0.26	−0.57 to −0.05
Time to 1 st oral liquid intake (days)		0.4 (0.3–0.7)	0.5 (0.3–0.8)	0.765	−0.01	−0.1 to 0.08
Time to 1 st oral solid intake (days)		0.9 (0.8–1.8)	1.1 (0.8–1.3)	0.797	−0.02	−0.24 to 0.19
Time to flatus (days)		1.1 (0.7–1.9)	1.3 (0.8–1.9)	0.285	−0.16	−0.45 to 0.13
Surgical site infection (SSI) within 30 days	Yes	0 (0%)	1 (1%)	0.317	0	NA
	No	95 (100%)	94 (99%)			
30-day readmission	Yes	2 (2%)	9 (9%)	**0.035**	0.22	0.05 to 1.03
	No	93 (98%)	86 (91%)			
Postoperative ileus incidence	Yes	5 (5%)	4 (4%)	0.705	1.33	0.3 to 5.97
	No	90 (95%)	91 (96%)			
Postoperative nausea/vomiting	Yes	53 (56%)	57 (60%)	0.564	0.85	0.48 to 1.49
	No	42 (44%)	38 (40%)			
Postoperative other complications	Yes	8 (8%)	9 (9%)	0.796	0.88	0.32 to 2.42
	No	87 (92%)	86 (91%)			
Total hospital costs ($1,000)		13.1 (11.5–15)	13.9 (12.3–15.5)	0.122	−0.7	−1.63 to 0.18

Matched data returned 190 patients for consideration (95 pairs of two pathway types). For continuous outcome measures, some paired observations were removed due to missing data. All outocomes were analyzed using 95 pairs of data except for time to 1^st^ oral liquid intake (89 pairs), time to 1^st^ oral solids (45 pairs), time to flatus (52 pairs) and total hospital costs (90 pairs). For continuous variables, the summary statistics are medians (1^st^, 3^rd^ quartiles), and the P-values were based pairwise Wilcoxon signed rank test. For categorical variables, the summary statistics are counts (%), and the P-values were based on McNemar test

*Bolded values are significant at *p* < 0.05

Pain scores and opioid consumption as measured by oral morphine equivalents were significantly lower in the ERAS cohort than in the traditional cohort at all the postoperative time points (0–1-hour, 1–24 h, and 24–48-hour) (Table 5).

**Table 5:** Patient pain scores and opioid consumption

Outcome	ERAS Pathway	Traditional care	*P*-value	Median difference (ERAS – Traditional)	95% CI
*Pain Score*					
Highest during stay	6 (5–8)	9 (7–10)	**< 0.001**	−2.5	−3 to −1.5
Highest during 0–1 h	5 (3–7)	7 (5–9)	**0.001**	−2	−3 to −1
Highest during 1–24 h	5 (4–7.5.5)	8 (7–10)	**< 0.001**	−3	−3.5 to −2
Highest during 24–48 h	4 (2–6)	6 (4–8)	**< 0.001**	−2	−3 to −1
*Postoperative oral morphine equivalents (OME*,* mg)*					
Total	67.5 (42.2–104)	102 (66–140.5.5)	**< 0.001**	−34.25	−49.75 to −19.25
0–1 h	4 (0–16)	12 (4–24)	**< 0.001**	−7.5	−11.5 to −4
1–24 h	36 (21–54.2.2)	51 (33–67.5.5)	**0.001**	−14.5	−23 to −6
24–48 h	20 (10–35)	37.5 (20–50)	**< 0.001**	−13	−21.5 to −6.25

All outcomes have 95 pairs of data, except highest pain score during stay (94 pairs), highest pain score 24–48 h (89 pairs), and OME 24–48 h (94) pairs). The summary statistics are medians (1^st^, 3^rd^ quartiles). The P-values were based on pairwise Wilcoxon signed rank sum test. All the P-values were significant

^a^Bolded values are significant at *p* < 0.05

^b^Pain scores based on the visual analog scale

^c^Opioid consumption in oral morphine equivalents (OME)

## Discussion

Minimally invasive approaches to renal surgery are playing an increasingly important role in urology. Robotic surgery has been shown to offer key benefits over both laparoscopic and open radical nephrectomy, and is associated with shorter LOS, fewer complications, and lower estimated blood loss. Similar results of decreased LOS have been observed in patients undergoing robotic partial nephrectomy compared to those undergoing laparoscopic and open approaches [11]. However, robotic approaches have limitations such as, longer operative time and high patient cost ([9]– [10]).

The results of our study support our primary hypothesis that implementation of an ERAS pathway can reduce hospital LOS in robotic nephrectomy procedures independent of demographic, preoperative (i.e. comorbidities) and intraoperative (i.e. nephrectomy type) factors. Our LOS results correlate with outcomes in LOS observed in smaller enhanced recovery studies in robotic nephrectomy procedures and other urological surgeries ([15, 20, 24–26]. Although a statistically significant difference was observed, in LOS, (2.2 vs. 2.3 days) it is debatable whether this represent a clinically significant difference and differences in time to discharge may be easily influenced by unaccounted variables such as method of transportation, prescription delivery/collection, nursing to patient discharge education.

When evaluating perioperative outcomes, patients in our study who received ERAS demonstrated a trend towards more rapid time to recovery milestones such as time to first oral liquid and solid intake, and time to first flatus. However, none of these variables reached statistical significance. Furthermore, many of these variables suffered from missing data. Similar observations of accelerated recovery milestones have also been reported in patients undergoing radical cystectomy in combination with ERAS [2, 17, 18, 27]. Within the field of robotics, data supporting the benefits of ERAS remain sparse; however, decreased time to ambulation and liquid intake have been observed in patients undergoing robotic prostatectomy [19, 20]. These findings are not entirely surprising as they are a key element of the ERAS protocol, which includes early postoperative mobilization and feeding. Although prior research has suggested faster return of bowel function in ERAS patients undergoing other urologic procedures, such as cystectomy, prostatectomy, and nephrectomy [2, 3, 25], our study did not observe a statistically significant difference in time to return of bowel function as measured by time to first flatus.

ERAS programs focus on perioperative measures to increase early gastrointestinal transit and prevent postoperative ileus (POI), which is considered a major driver of LOS [27, 28]. Our study did not reveal a significant decrease in POI but did demonstrate statistically significant differences between groups that may influence length of stay to a greater degree than bowel function, such as postoperative pain control. Our ERAS cohort demonstrated significantly lower pain scores and opioid consumption compared to those undergoing traditional nephrectomy, thus supporting the implementation of an ERAS protocol that combines a comprehensive multimodal non-opioid strategy allowing for improved patient analgesia and patient comfort.

After implementation of the ERAS pathway, we observed a reduction in both postoperative pain scores and opioid analgesic requirements at all postoperative time points compared with those receiving traditional care. These findings are consistent with previous ERAS studies demonstrating lower pain scores and postoperative opioid use in patients undergoing robotic-assisted prostatectomy (RARP) and laparoscopic hand-assisted donor nephrectomy with ERAS [6, 15, 24]. Furthermore, other studies have suggested that the use of less invasive approaches (DaVinci SP), can predict opioid-free status following RARP [16]. A comprehensive ERAS pathway involving various opioid-sparing analgesic techniques can significantly lower pain scores and opioid use throughout the patient’s perioperative journey.

The opioid epidemic has emerged as one of public health’s top priorities with opioid related deaths having increased by 300% in the past decade [14]. Perioperative opioid prescribing is believed to play a contributing role in a complex ecosystem that is the opioid epidemic. Per capita opioid prescription in the United States exceeds the global average and perioperative opioid prescription is a clear contributor to the opioid pool with greater than 300 million operations being performed annually [12]. Additionally, prior studies have demonstrated increased odds of developing persistent opioid use following surgery related to the amount of opioids prescribed [13]. While our study does not evaluate opioid prescriptions, it does demonstrate lower in-hospital opioid requirements, which may be the first step in optimizing and reducing postoperative opioid prescription.

Based on our study, little remains known about which modalities of opioid-sparing techniques have the greatest impact on attenuating postoperative pain and discomfort in robotic nephrectomy and further studies are warranted to ascertain which components of these multimodal pathways are of the greatest value in contributing to patient analgesia.

For patients undergoing robotic partial, or non-partial nephrectomy, ERAS did not appear to confer a significant benefit in terms of reduction in postoperative complications. There was, however, a statistically significant difference in 30-day readmission favoring ERAS over traditional care, with patients in the ERAS and traditional groups experienced readmission on average 11.8 and 12.5 days after discharge, respectively. This was not observed in similar studies implementing ERAS pathways in robotic and laparoscopic nephrectomies, perhaps due to their small sample size [24, 25, 29]. These findings exist alongside data highlighting that patients undergoing minimally invasive radical and partial nephrectomies already experience decreased complication, readmission, and mortality rates when compared to open surgery [8, 21–23]. Thus, ERAS may only provide marginal benefit over traditional care within minimally invasive surgery regarding postoperative complications and readmission or perhaps would require much larger sample sizes to detect differences.

Although not statistically significant, we observed an objective reduction in hospital costs with an $800 reduction in hospital costs per patient in the ERAS cohort. This is important when considering value-based care and bundled payments, along with increased expenses in economic healthcare. Our observations are in line with other cost analysis of surgical services that have developed enhanced recovery programs. We attribute that our cost-saving was likely associated with the lower length of stay observed in the ERAS group, as has been seen with many other surgical services that have implemented enhanced recovery pathways into their surgical programs [24, 27, 30].

Our study had several limitations. This was a single-center retrospective study and thus represents an inferior level of evidence compared to prospective studies. Data collection was limited to what was retrieved from the electronic health record, and variability in the accuracy of measuring the time to recovery milestones was inevitable. Certain patient recovery milestones such as time to first solid intake and time to first flatus were missing a disproportionate amount of data contributing to fewer matched pairs in final analysis. We acknowledge missing data may have been due to variability and inconsistency in patient charting or differences in provider preference for discharge appropriateness (providers discharging patients on clear liquid diet thus not allowing in-hospital achievement of oral solid intake or not requiring patients to pass gas as a discharge pre-requisite thus failing to capture this recovery milestone). This limitation is a source of measurement bias but is critical to understanding the hospital course and milestones that direct patient discharge. Future prospective studies with standardized postoperative pathways and discharge pre-requisites are needed to reliably capture these patient-reported outcomes (PROs). Additionally, our study assumes no administration of local analgesia or non-opioid analgesics in our cohort undergoing traditional care, despite the likelihood that some patients within this cohort might have received local analgesia or non-opioid analgesics at the time of surgery. However, even if there was consistent avoidance of these analgesics in the control group, this would have further supported that a standardized enhanced recovery pathway significantly contributes to better patient pain control and shorter LOS. Differences in demographic and clinical variables were observed between the groups; however, their possible confounding nature was accounted for with multivariate logistic regression. Differences between groups were mitigated by propensity score matching; however, some paired observations were excluded due to missing data. Finally, while our study did demonstrate significantly reduced postoperative opioid consumption and greater pain control in patients undergoing ERAS, this was limited to in-hospital administration of opioids, and little is known about postoperative opioid prescription patterns and ambulatory outpatient consumption.

## Conclusion

Our review demonstrates that patients undergoing robotic renal surgery with a comprehensive perioperative ERAS pathway experience significant benefits in terms of LOS and reduction in readmission rates. This study also contributes to a growing body of literature supporting ERAS pathways as a promising protocol for optimizing patient comfort postoperatively and reducing opioid consumption which has significant implications in the context of the current opioid epidemic. Further prospective studies across a variety of minimally invasive and robotic surgeries are needed to evaluate the efficacy and benefits of specific ERAS elements in improving postoperative patient outcomes. Additional studies evaluating long-term quality of life would further determine the impact of ERAS in patients undergoing minimally invasive urologic surgery.

## Data Availability

No datasets were generated or analysed during the current study.
